# Effect and prognostic significance of the KAI1 gene in human gastric carcinoma

**DOI:** 10.3892/ol.2015.3604

**Published:** 2015-08-12

**Authors:** JING GUO, KAI-XI FAN, LI XIE, JIA-JIA XIAO, KAI CHEN, LI-NA HUI, ZHONG-FA XU

**Affiliations:** 1 Department of Oncology, Affiliated Hospital of Shandong Academy of Medical Sciences, Jinan, Shandong 250031, P.R. China; 2 Basic Laboratory, Shandong Cancer Hospital and Institute, Jinan, Shandong 250117, P.R. China; 3 Department of Neurology, Shanghai First People's Hospital Affiliated to Shanghai Jiao Tong University, Shanghai 200080, P.R. China; 4 Department of Thoracic Surgery, The Third Affiliated Hospital of Kunming Medical University, Kunming, Yunnan 650118, P.R. China

**Keywords:** gastric cancer, KAI1 gene, anti-oncogene, gene transfection, prognosis

## Abstract

The present study aimed to explore the effect and mechanism of the Kangai 1 (KAI1) gene in regulating the migration and invasion of gastric carcinoma cells, and the prognostic significance of this gene in gastric cancer patients. Immunohistochemistry and *in situ* hybridization were used to investigate the role of KAI1 in the progression and prognosis of gastric cancer. The pEGFP-N1-KAI1 plasmid was transfected into human gastric carcinoma SGC7901 cells using liposomes. The effect of transfection with the KAI1 gene was measured using a reverse transcription-semi-quantitative polymerase chain reaction (RT-sqPCR) assay. The Transwell chamber assay was used to study the metastatic and invasive ability of SGC7901 cells. Gastric cancer metastasis-associated genes, including hypoxia-inducible factor (HIF)-1α, matrix metalloproteinase (MMP)-2, MMP-9, basic fibroblast growth factor (bFGF), and urease plasminogen activator (uPA) were measured by RT-sqPCR prior to and following transfection with the KAI1 gene. The expression of KAI1 protein and mRNA was associated with the differentiation degree of gastric cancer, presence of lymph node metastasis, tumor-node-metastasis stage, depth of invasion and the survival time of patients. The migratory and invasive abilities of SGC7901 cells were significantly decreased subsequent to transfection with the KAI1 gene, and the expression of bFGF and uPA was downregulated. It was concluded that the tumor suppressor gene KAI1 inhibits the migration and invasion of gastric carcinoma cells, possibly by suppressing the expression of uPA. Patients that expressed KAI1 may demonstrate an improved prognosis.

## Introduction

Gastric cancer is the second highest cause of cancer-associated mortality worldwide. Despite the advances in diagnosis and treatment, the prognosis of patients with gastric cancer remains poor. The median survival time of patients with advanced gastric cancer is ≤10 months, and only 10–20% of patients survive >5 years.

Kangai 1 (KAI1), also termed cluster of differentiation (CD)82, is a tumor metastasis suppressor that was first identified as a metastasis suppressor for prostate cancer. KAI1 is located on human chromosome 11p11.2 (1) and is ~80 kb in length, containing 10 exons and 9 introns. This gene encodes a 267-amino acid protein that is a member of the transmembrane 4 superfamily (TM4SF). KAI1 has been found to be downregulated in numerous types of human cancers, including prostate, breast and ovarian cancers. A previous study has identified that p53 plays a role in the positive regulation of the expression of the KAI1 gene and may activate KAI1 through the consensus binding sequence in the promoter (2). In addition, the major cause of the poor prognosis of patients with gastric cancer is the reduction in the expression of these two proteins (3). It has also been reported that the expression of KAI is downregulated in advanced cancer, and more so in metastatic cancer (4–6). Consequently, KAI1 expression plays an important role in cancer progression and may also be a potential target for the inhibition of cancer metastasis. In order to detect the role of KAI1 in the progression and prognosis of gastric cancer, immunohistochemistry and *in situ* hybridization were used in the present study to evaluate KAI1 expression in various stages of gastric cancer.

At present, no specific studies have been conducted to investigate the effects and mechanisms of the KAI1 gene on the migration and invasion of gastric carcinoma cells. To detect these aspects, the pEGFP-N1-KAI1 plasmid was transfected into the gastric carcinoma SGC7901 cells through liposomes in the present study.

## Materials and methods

### 

#### Patients

Tissue specimens obtained from 128 patients with gastric adenocarcinoma that underwent resection at the Shandong Cancer Hospital (Jinan, Shandong, China) between January 2007 and April 2009 were used in the present study. The patients consisted of 81 males and 47 females, aged between 30 and 74 years (median, 48 years). The inclusion criteria for the present study were as follows: Complete surgical R0 resection of the primary tumor; pathologically confirmed diagnosis of gastric adenocarcinoma; no chemotherapy or radiotherapy administered; and the absence of secondary malignancies. All patient records contained complete clinical, pathological and follow-up data. Normal gastric mucosa tissue (≤5 cm) adjacent to the tumor was excised and confirmed to be tumor-free by pathological analysis.

Tumor histology was determined according to the criteria provided by the World Health Organization (7). The pathological tumor-node-metastasis (TNM) stage was assessed according to the Unified International Gastric Cancer Staging Classification System, as incorporated in the UICC TNM classification manual (8). The clinical outcome of the patients was followed up from the date of surgery to either the date of mortality or April 20, 2014, resulting in a follow-up period of 1–60 months (mean, 40 months). The present study was conducted in accordance with the Declaration of Helsinki (9), and the Ethics Committee of the Affiliated Hospital of Shandong Academy of Medical Sciences (Jinan, Shandong, China) approved the present experimental protocols. Written informed consent was obtained from all patients.

#### Immunohistochemistry

The tissue sections were conventionally dewaxed, hydrated and subjected to antigen repair with EDTA. The monoclonal mouse anti-human KAI1/CD82 antibody (cat no. 564341; BD Biosciences, San Jose, CA, USA) was diluted at 1:200. The immunohistochemical staining was performed using the of streptavidin-peroxidase two-stage method, according to the instructions of the kits (Fuzhou Maixin Biotech Co., Ltd., Fuzhou, Fujian, China). Negative controls were stained following the same procedure, with the exception that the primary antibody was replaced with PBS. The KAI1-positive tissue provided by Fuzhou Maixin Biotech Co., Ltd. was used as a positive control.

The staining intensity and percentage of cells stained for KAI1 expression were evaluated in a blind manner by three pathologists simultaneously, and a consensus was reached for each score. Cells positive for the expression of KAI1 were considered to be cells with brown plasma membranes and cytoplasm. The presence of KAI1 expression was assessed through the ratio of stained to non-stained cells. At least nine visual fields were observed for each section under a high power lens (H600L; Nikon, Tokyo, Japan). The staining intensity was judged based on the ratio of KAI1-positive to total cell numbers observed in the visual field. Sections with ≤10% KAI1-positive cells were considered to not express KAI1 and sections with >10% KAI1-positive cells were considered to express KAI1.

#### In situ hybridization

The mRNA sequence of the KAI1 gene was retrieved from the National Center for Biotechnology Information database (U.S. National Library of Medicine, Bethesda, MD, USA). The oligonucleotide probe sequences were designed using Primer3 software (Whitehead Institute for Biomedical Research, Cambridge, MA, USA) as follows: 5′-CAGCCTTTCTGTGAGGAAGG-3′ (800–819 bp); 5′-GATGGTCCTGTCCATCTGCT-3′ (983–1,002 bp); and 5′-GCAGTCACTATGCTCAT-3′ (438–454 bp) (Sangon Biotech Co., Ltd., Shanghai, China). The primers were marked by digoxin. The tissue sections were conventionally dewaxed and underwent gradient alcoholic dehydration. The slides were incubated in 3% H_2_O_2_ at room temperature for 10 min, and were then digested using Proteinase K, diluted in 3% saline sodium citrate, at 37°C for 20 min. The *in situ* hybridization was performed according to the instructions for the kits (Roche, Basel, Switzerland). Blank controls were operated following the same procedure, but without the probe. The expression of KAI1 was indicated by *in situ* hybridization as clear yellow brown granular material, which was located on the cell membrane. The KAI1 staining intensity was scored as follows: 0, absent; 1, weak; 2, moderate; and 3, strong. The percentage of KAI1-positive cells was scored into four categories, as follows: 1, 0–10%; 2, 11–30%; 3, 31–60%; and 4, 61–100%. The sum of these two scores was classified as follows: 1–3, absent; 4–5, positive; and 6–7, strongly positive.

#### Eukaryotic expression plasmid vector, cell culture, plasmid transfection and reverse transcription-semi-quantitative polymerase chain reaction (RT-sqPCR) assay

pEGFP-N1 is a eukaryotic expression plasmid without the objective gene, and this plasmid possesses a selectable marker gene for G418 resistance. The eukaryotic expression plasmid vector was supplied by Proteintech Group Inc. (Chicago, IL, USA).

#### Cell culture

The SGC7901 cell line was obtained from the Cell Bank of the Chinese Academy of Sciences (Shanghai, China). The cells were cultured in Dulbecco's modified Eagle's medium (DMEM; Sigma-Aldrich, St. Louis, MO, USA) containing 10% fetal bovine serum (FBS; HyClone, Logan, UT, USA) at 37°C in an incubator with a humidified 5% CO_2_ atmosphere.

#### Plasmid transfection

Once the cells had reached 70–90% confluency, the pEGFP-N1-KAI1 plasmid was transfected into the SGC7901 cells using Lipofectamine 2000 (Invitrogen, Carlsbad, CA, USA), in accordance with the manufacturer's instructions. The vector control plate was transfected with the pEGFP-N1 plasmid, and cells without transfection acted as a blank control.

#### RT-sqPCR assay

The effect of KAI1 gene transfection was measured using an RT-sqPCR assay. Total RNA with isolated using TRIzol (Invitrogen, Carlsbad, CA, USA) using an RNeasy Mini kit (cat no. 74104; Qiagen, Dusseldorf, Germany) and cDNA was generated by reverse transcription, according to the instructions of the Reverse Transcription System kit (Promega, Madison, WI, USA). The primers used for KAI1 PCR were as follows: Forward, 5′-CCCCAAGTACTGAGGCAGC-3′, and reverse, 5′-AACCACAGAACAGCCAGGG-3′. This generated a 217-bp product (1040–1256 bp) (Fig. 2A). The PCR mixture contained 5 µl 2X HiFi PCR Master Mix, 0.5 µl forward primer (2 µM), 0.5 µl reverse primer (2 µM), 3.5 µl RNase-free double-distilled H_2_O, and 0.5 µl cDNA template. The PCR conditions were as follows: 95°C for 3 min; 30 cycles at 94°C for 20 sec, 55°C for 20 sec and 72°C for 20 sec; and 72°C for 5 min.

#### Cell migration and invasion assays

A Transwell chamber assay was used to perform the cell migration analysis. The cells were fasted for 24 h with serum-free medium DMEM containing 0.1% bovine serum albumin (BSA), and then trypsinized and resuspended with medium to a density of 1×10^6^/ml. The three groups of cells were seeded in the upper chamber of the Transwell insert, and 600 µl DMEM containing 10% FBS was added to the lower chamber. The cells were cultured at 37°C in a humidified 5% CO_2_ incubator for 24 h, the inserts were washed with PBS. A cotton swab was used to remove adherent cells on the inner side of the upper chamber membrane. The upper chamber was then dried naturally and stained with 0.5% hematoxylin. Six visual fields of each insert were randomly counted under an upright light microscope (BX51; Olympus, Tokyo, Japan), and the average cell number was calculated.

#### Cell invasion assay

The Transwell chamber invasion assay was performed to investigate the role of KAI1 in gastric cancer cells. Laminins (20 µg/ml; EMD Millipore, Billerica, MA, USA) were diluted in serum- and BSA-free DMEM and were then added to a 24-well plate at 0.95 ml/well (10 µg/cm^2^). This process was repeated 3 times. The plate was then incubated at 4°C overnight. The subsequent experimental steps were in accordance with the cell migration assay.

#### RT-sqPCR

Total RNA was obtained using TRIzol extraction (Invitrogen, Carlsbad, CA, USA) and reverse transcribed into complementary DNA using the kit (Takara RT-PCR), according to the manufacturer's instructions. RT-sqPCR was performed by using the Roche Cobas 4800 System (Roche). The sequences of the HIF-1α^x^, MMP-2^x^, MMP-9^x^, bFGF^x^ and uPA primers are described in Table I.

#### Statistical analysis

The PEMS 3.1 software (Jingyuan Guangzhou Pharmaceutical Research Ltd., Guangzhou, China) was used for the statistical analysis. All data were expressed as the mean ± standard deviation from at least three independent experiments. The KAI1 staining level in tissues from various stages of gastric cancer was compared using the χ^2^ test. The Kaplan-Meier survival curve and log-rank test were used to analyze the association between KAI1 expression and patient survival. The correlation analysis was performed using Spearman's rank correlation coefficient test, and the inspection level was α=0.05. P<0.05 was considered to indicate a statistically significant difference.

## Results

### 

#### KAI1 protein expression in gastric cancer tissue and the correlation with clinical pathology

The KAI1 protein was found to be expressed in normal gastric mucosa tissues, with an expression rate of 22% in gastric cancer tissue, and the difference between the expression rate of KAI1 in cancer and normal tissues was statistically significant (χ^2^=24.382; P=0.000). The expression of KAI1 was associated with the differentiation degree of gastric cancer tumors (Table II). The deeper the degree of gastric cancer invasion, the lower the rate of KAI1 expression (Table III). Correlation analysis was used to further detect the association between the various clinical stages and the positive expression of the KAI1 protein. The present results indicated a negative correlation between the clinical stages and the positive expression of KAI1 (r=-0.9890; P=0.0110; Table III).

#### KAI1 mRNA expression in gastric cancer tissue and the correlation with clinical pathology

KAI1 mRNA expression was present in all normal gastric mucosa samples and in 40 out of 128 gastric carcinoma tissue samples, which was a statistically significant difference (P=0.0001). There was no significant difference (P>0.05) between the presence of KAI1 mRNA expression and the tumor location, or between the age and gender of the patients. The rate of KAI1 mRNA expression in the superior differentiation group, which consisted of well- and moderately-differentiated adenocarcinoma, was increased compared with the expression in the inferior differentiation group, which consisted of poorly-differentiated and mucinous adenocarcinoma and signet-ring cell carcinoma (P<0.05). Correlation analysis was used to analyze the association between the depth of invasion and KAI1 mRNA expression, and there was a significant negative correlation between these variables (r=-0.9558; P=0.044 2). Similarly, there was a significant negative correlation between the TNM stage and KAI1 mRNA expression (r=-0.9891; P=0.0109). The rate of KAI1 mRNA expression in gastric cancer patients with lymph node metastasis was markedly decreased compared with the rate in gastric cancer patients without lymph node metastasis, and the difference was statistically significant (P<0.05; Table IV).

#### Correlation between KAI1 expression and prognosis in patients with gastric cancer

**Figure 1.**
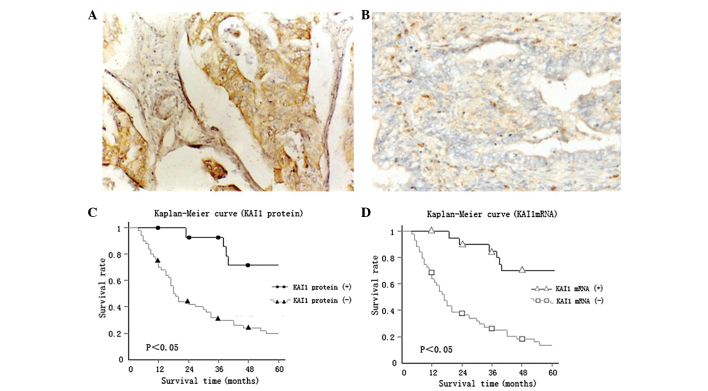
KAI1 protein and mRNA expression in gastric cancer tissue and the correlation with the prognosis of gastric cancer patients. (A) Positive staining for the expression of the KAI1 protein was brown in color and diffusely distributed, with staining being located in the cytoplasm and plasma membrane (SP 400). (B) KAI1 mRNA positive staining was mainly located in cytoplasm, and cells expressing KAI1 were brown granular with clear location. (C) Effect of KAI1 protein expression on the survival time in gastric cancer patients. In total, 28 gastric cancer patients demonstrated KAI1 expression and 100 gastric cancer patients did not express KAI1. The survival time of the group that expressed the KAI1 protein was significantly longer compared with the group that did not express the KAI1 protein (P<0.05). (D) Effect of KAI1 mRNA expression on the survival time in gastric cancer. In total, 40 gastric cancer patients expressed KAI1 mRNA and 88 gastric cancer patients did not express KAI1 mRNA. The survival time of the group that expressed KAI1 mRNA was significantly longer compared with the group that did not express KAI1 mRNA (P<0.05). KAI1, Kangai 1.

The result of Kaplan-Meier analysis indicated that the survival time of the group that expressed KAI1 was significantly longer compared with the group that did not express KAI1. The log-rank test indicated that the difference between the two groups was statistically significant (χ^2^=11.523; *v*=1; P<0.05; Fig. 1C).

In addition, the difference between the five-year survival rates of the groups expressing and not expressing the KAI1 protein was statistically significant (P<0.05; Table V). Thus, patients expressing the KAI1 protein demonstrated an improved prognosis.

#### KAI1 mRNA expression in gastric cancer tissue and its correlation with the prognosis of gastric cancer patients

The statistical results of Kaplan-Meier indicated that the survival time of the group expressing KAI1 mRNA was longer compared with the group without KAI1 mRNA expression, and the difference was statistically significant (P<0.05; Fig. 1D). However, the difference between the five-year survival rate of the group expressing KAI1 mRNA and group without KAI1 expression was statistically significant (P<0.05; Table VI). Therefore, patients demonstrating KAI1 mRNA expression had an improved prognosis, and this result was consistent with the aforementioned conclusion.

#### Transfection of SGC7901 cells

**Figure 2.**
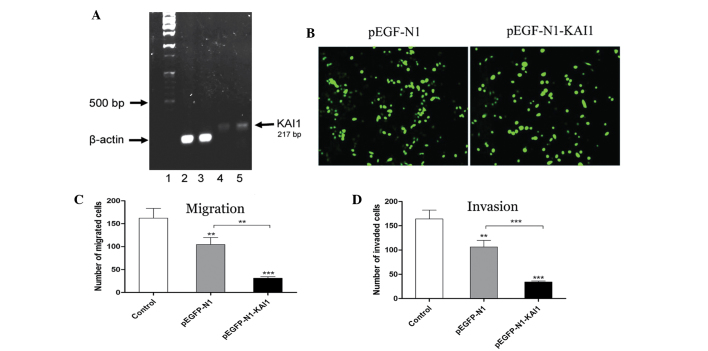
Findings of the RT-sqPCR, transfection, migration and invasion assays. (A) The pEGFP-N1-KAI1 plasmid was transfected into human gastric carcinoma SGC7901 cells by liposome. KAI1 was clearly overexpressed in pEGFP-N1-KAI1 transfected cells (lane 5) compared to the control cells (lane 4). Lanes 1, 100 bp+1kb ladder; lane 2, β-actin expression in pEGFP-N1-transfected SGC7901 cells; lane 3, β-actin expression in pEGFP-N1-KAI1-transfected SGC7901 cells; lane 4, KAI1 expression in pEGFP-N1-transfected SGC7901 cells; and lane 5, KAI1 expression in pEGFP-N1-KAI1-transfected SGC7901 cells. KAI1 was clearly more highly expressed in lane 5 compared with lane 4. (B) Fluorescent expression of pEGFP-N1-KAI1 and pEGFP-N1 in transfected SGC7901 cells, revealing that transfection with the KAI1 gene inhibited the migration and invasion activity of SGC7901 cells. (C) The invasion activity of pEGFP-N1-KAI1 cells was significantly downregulated. (D) The migratory activity of pEGFP-N1-KAI1 cells was significantly decreased. RT-sqPCR, reverse transcription-semi-quantitative polymerase chain reaction; KAI1, Kangai 1.

To examine the effect of the expression of KAI1 on the SGC7901 cells, gastric cancer SGC7901 cells were transiently transfected with the pEGFP-N1-KAI1 and pEGFP-N1 plasmids. Electrophoresis of the RT-sqPCR products revealed that KAI1 was evidently overexpressed in the cells transfected with pEGFP-N1-KAI1 compared with the cells transfected with pEGFP-N1 (Fig. 2A). Fluorescent expression was observed in the cells transfected with pEGFP-N1-KAI1 and those transfected with pEGFP-N1 (Fig. 2B).

#### Cell migration and invasion

The migratory and invasive ability of SGC-7901 cells was detected using a Transwell assay. The number of cells that had traversed the membrane was counted subsequent to transfection for 48 h. Compared with the cells transfected with the pEGFP-N1 plasmid, the migratory and invasive activity of the cells transfected with the pEGFP-N1-KAI1 plasmid was significantly decreased (Fig. 2C and D).

#### Expression of metastasis-associated genes in gastric cancer cells

RT-sqPCR was used to measure the effects of KAI1 transfection on the expression of metastasis-associated genes in SGC7901 cells. Table VII reports that the expression of HIF-1α was slightly increased following transfection with the KAI1 expression plasmid. The expression of MMP-9 was slightly reduced, but this effect was not statistically significant (P>0.05). The expression of MMP-2 was significantly increased in gastric carcinoma SGC7901 cells by KAI1 expression, whereas the expression of uPA was significantly downregulated. The expression of bFGF was also significantly decreased (P<0.05).

## Discussion

KAI1 was first identified as a metastasis suppressor in prostate cancer (1). Subsequent studies demonstrated that this gene suppresses invasion and metastasis in various cancers during disease progression (4,10). The present study used immunohistochemistry and *in situ* hybridization (MK2158; Wuhan Boster Biological Technology Co., Ltd., Wuhan, China) to examine 16 cases of well-differentiated adenocarcinoma, 28 cases of moderately-differentiated adenocarcinoma, 66 cases of poorly-differentiated adenocarcinoma, 10 cases of mucinous adenocarcinoma and 8 cases of signet-ring cell carcinoma. In addition, the effects of KAI1 expression on the migration and invasion of gastric cancer cells were investigated, as migration and invasion are the critical steps in cancer progression. The results of the present study demonstrated that KAI1 staining occurs mainly in the cytoplasm (Fig. 1A), which is consistent with the findings of previous studies (4,10,11).

Statistical analysis of the positive rate of KAI1 revealed a significant reduction in cytoplasmic KAI1 expression in internal gastric cancer compared with superficial gastric cancer, indicating that reduced KAI1 expression may be critical for the progression of gastric cancer. The present data revealed that decreased KAI1 expression was significantly associated with the presence of lymphatic metastasis, TNM stage, patient survival time, depth of invasion and the degree of gastric cancer differentiation. The results also indicated that reduced KAI1 expression was not involved in distant metastasis, but significantly stimulated gastric cancer cell migration (Fig. 2C) and invasion (Fig. 2D), the two important events in the process of tumor progression into metastasis (12). This indicates that KAI1 is a potent gastric cancer metastasis suppressor. In addition, analysis of the survival curves in the present study revealed that the expression of KAI1 was positively associated with the survival of patients, which is consistent with the conclusions from studies performed on malignant melanoma (11), non-small-cell lung cancer (13) and prostate cancer (14).

The KAI1 protein plays an important role in signaling pathways, as it mediates the signal transduction between cells and the surrounding environment, which affects the movement and differentiation of cells and ultimately inhibits the invasion and metastasis of tumor cells. KAI1 may regulate the infiltration and metastasis of tumors through the following four mechanisms. First, KAI1 binds with integrin to form a complex and affects cell adhesion by regulating the functions of integrin. Second, the protein promotes adhesion between tumor cells by acting on the signal pathway of Src kinase (15). Third, KAI1/CD82-associated surface protein is involved in the KAI1 signaling pathway (16). Fourth, the inhibition of KAI1 is mediated by numerous other signaling pathways and factors, including FAK, Rac and GTPase (17,18).

In addition, KAI1 is a highly glycosylated protein, and a deficiency in the glycosylation process leads to a lack of KAI1 function, without affecting the expression of the protein (13). The signaling pathway mediated by INF may activate nuclear factor-κB and increase the expression of KAI1 (19).

In the present study, the expression of HIF-1α, MMP-2, MMP-9, bFGF and uPA was detected in order to further reveal the underlying mechanism of the function of the KAI1 gene in gastric cancer. The current study identified that the inhibitory effect of KAI1 expression on the cell migration and invasion of gastric cancer was not mediated by HIF-1α, MMP-2 and MMP-9, but may be associated with the reduction in uPA and bFGF expression.

bFGF may promote angiogenesis and degrade the extracellular matrix by upregulating the expression of MMPs (20). bFGF may also directly induce the tumor cells to secrete a variety of protein decomposing enzymes and collagenases, in order to promote tumor metastasis and invasion. Notably, a previous study revealed that uPAR ligation enhanced the migration of human lung fibroblasts by recruiting α5β1 integrin and lipid rafts through caveolin-Fyn-Shc signaling (21). This mechanism may be applicable to the present study. It was therefore hypothesized that in the current study, uPAR ligation increased the migration of gastric cancer cells, possibly through the recruitment of α5β1 integrin complexes, alongside the activation of the lipid raft-localized caveolin-Fyn-Shc pathway. This, in turn, led to a hypermigratory phenotype. As a result, the reduced expression of uPA may limit the activation of this pathway and therefore, decrease the migratory ability of cancer cells. However, the underlying specific mechanism requires additional investigation.

The present study demonstrated that KAI1 may be used as a prognostic marker in human gastric cancer, which is not consistent with the findings of a previous study by Knoener *et al* (22). This is possibly due to the various characteristics of the respective studies, including the ethnicity, experimental specimens, surgical technology and reagents. And the main difference is specimen. As we all know, specimens kept for too long might lead to an antigen loss. Therefore, we could collect more recent cases to detect whether KAI1 is a prognostic marker or not.

Furthermore, the experimental data revealed that the expression of MMP-2 significantly increased following transfection with the KAI1 gene, which was not consistent with the anti-metastatic role. This discrepancy may be due to the initiation of malignant tumor development being an extremely complicated process that involves multiple anomalies of signaling pathways, including the induction of apoptosis, regulation of the cell cycle and inhibition of tumor cell adhesion. Proto-oncogenes, anti-oncogenes and multiple other factors participate in this process. The association between these factors is complex, and the effect may be negative, positive or modulatory, and in various forms. In addition, the association may change with the progression of tumors. Anti-oncogenes may not inhibit all the cocarcinogens. Therefore, it is not notable that the expression of MMP-2 significantly increased subsequent to transfection with the KAI1 gene. This result also indicated that the inhibitory effect of KAI1 may not be mediated by MMP-2.

The present results demonstrated clearly that the invasive ability of gastric cancer SGC7901 cells was significantly decreased subsequent to transfection with the KAI1 gene. In addition, the KAI1 gene may suppress the migration and invasion of SGC7901 cells by reducing the expression of uPA and bFGF. Therefore, the present data indicated that the expression level of KAI1 protein may affect the migration and invasion ability of gastric cancer cells. Upregulation of KAI1 gene expression in gastric cancer cells may represent an important direction to inhibit the invasion and metastasis of gastric cancer, which requires additional investigation.

In summary, the present study confirmed that KAI1 plays an important role in the inhibition of metastasis and invasion of gastric carcinoma, and downregulated KAI1 expression was significantly associated with advanced gastric cancer and a poor five-year survival rate in patients. The present study provides a theoretical and experimental basis for the investigation and clinical application of KAI1. It was concluded that KAI1 may be used as a potential therapeutic target and is a promising prognostic marker for gastric cancer.

## Figures and Tables

**Table I.** Polymerase chain reaction primers for HIF-1α, MMP-2, MMP-9, bFGF, uPA and β-actin.

Gene	Direction	Primer sequence (5′-3′)
HIF-1α	F	AGCCAGACGATCATGCAGCTACTA
	R	TGTGGTAATCCACTTTCATCCATTG
MMP-2	F	TGTCGCCCCCAAAACGGACA
	R	ATGCTCCCAGCGGCCAAAGT
MMP-9	F	TGCTGGGCTGCTGCTTTGCT
	R	CGGGCAAAGGCGTCGTCAAT
bFGF	F	GAACGGGGGCTTCTTCCT
	R	CCCAGTTCGTTTCAGTGCC
uPA	F	TGAGCGACTCCAAAGGCAGCA
	R	TGAAGCAGTGTGTGGCGCTGA
β-actin	F	GGCATCGTGATGGACTCCG
	R	GCTGGAAGGTGGACAGCGA

F, forward; R, reverse; HIF-1α, hypoxia-inducible factor; MMP, matrix metalloproteinase; bFGF, basic fibroblast growth factor; uPA, urease plasminogen activator.

**Table II.** Association between KAI1 expression and the differentiation of gastric cancer tumors.

Differentiation	Total, n	KAI1-positive, n	Occupancy, %	χ^2^ value	P-value
Superior	44	18	40.9	5.5110	0.0189
Inferior	84	10	11.9		

Superior, well-differentiated and moderately-differentiated adenocarcinoma; inferior, poorly-differentiated and mucinous adenocarcinoma, and signet-ring cell carcinoma; KAI1, Kangai 1.

**Table III.** Association between KAI1 expression and other clinicopathological features of gastric cancer.

Clinicopathological feature	Total, n	KAI1-positive, n	Positive rates of KAI1, %	χ^2^ value	P-value
Depth of invasion					
Mucosa and submucosa	16	12	75.0	16.9004	0.0007
Muscular layer	48	10	20.8		
Serosa	44	6	13.6		
Out of serosa	20	0	0.0		
Lymphatic metastasis					
Yes	98	12	12.0	9.0682	0.0026
No	30	16	53.0		
Distant metastasis					
Yes	12	0	0.0	0.7104	0.3993
No	116	28	24.0		
TNM stage					
I	32	14	43.8	4.3881	0.0362
II	40	10	25.0		
III	44	4^a^	9.1		
IV	12	0	0.0		

a P<0.05 vs. stage I. The difference between the TNM staging was statistically significant (χ^2^=8.3782; P=0.0388). Analysis also indicated a significant difference in the KAI1-positive rate between stages I and III. Combining stage I and II into one group and stage III and IV into another group, there was significant difference in the KAI1 positive rate between the two new groups (χ^2^=4.8820, P=0.0271). KAI1, Kangai 1; TNM, tumor-node-metastasis.

**Table IV.** KAI1 mRNA expression in gastric cancer tissue and its correlation with clinical pathology.

		KAI1 mRNA expression			
				
Clinicopathological factor	Total, n	Present, n	Rate, %	χ^2^ value	P-value
Histological classification					
Superior differentiation	44	24	54.5	7.5416	0.0060
Inferior differentiation	84	16	19.0			
Depth of invasion					
T_1_	16	12	75.0		
T_2_	48	18	37.5	2.0497	0.1522
T_3_	44	8	18.2	5.8058	0.0131^a^
T_4_	20	2	10.0	5.4029	0.0201^a^
Lymphatic metastasis					
Yes	98	16	16.3	18.8097	0.0000
No	30	24	80.0			
TNM staging					
I	32	20	62.5		
II	40	14	35.0	1.7067	0.1914^b^
III	44	6	13.6	7.7757	0.0053^b^
IV	12	0	0.0	4.5852	0.0322^b^

a Compared with group T_1_.

b Compared with stage I. KAI1, Kangai 1.

**Table V.** Association between KAI1 expression and the survival time of patients.

		Expression of KAI1					
				
Survival time, years	Total, n	Present, n	Occupancy, %	Absent, n	Occupancy,%	χ^2^ value	P-value
>5	40	20	71	20	20	42.4261	0.000
<5	88	8	29	80	80		

KAI1, Kangai 1.

**Table VI.** The association between KAI1 mRNA expression and the survival time of patients.

		Expression of KAI1 mRNA					
				
Survival time, years	Total, n	Present, n	Occupancy, %	Absent, n	Occupancy, %	χ^2^ value	P-value
>5	40	28	70	12	13.6	19.1733	0.0007
<5	88	12	30	76	86.4		

KAI1, Kangai 1.

**Table VII.** Effect of KAl1 gene transfection on the expression of genes associated with gastric cancer metastasis.

	KAI1 expression		
		
Gene	Present	Absent	Fold change in expression
HIF-1α	1.7260	1.6700	1.034
MMP-2	7.9120	0.2715	28.76
MMP-9	1.2210	1.2740	0.958
bFGF	0.4565	0.5805	0.786
uPA	0.1621	7.5990	0.021

KAI1, Kangai 1; HIF-1α, hypoxia-inducible factor; MMP, matrix metalloproteinase; bFGF, basic fibroblast growth factor; uPA, urease plasminogen activator.
